# Effect of Total Replacement of Soya Bean Meal by Whole Lupine Seeds and of Gender on the Meat Quality and Fatty Acids Profile of Growing Rabbits

**DOI:** 10.3390/foods11162411

**Published:** 2022-08-11

**Authors:** Cristina M. Guedes, Mariana Almeida, Maude Closson, Sofia Garcia-Santos, José M. Lorenzo, Rubén Domínguez, Luís Ferreira, Henrique Trindade, Severiano Silva, Victor Pinheiro

**Affiliations:** 1 Veterinary and Animal Research Centre (CECAV), Associate Laboratory for Veterinary and Animal Science (AL4AnimalS), University of Trás-os-Montes e Alto Douro, Quinta de Prados, 5000-801 Vila Real, Portugal; 2 Higher Institute of Agronomy, University of Lisbon, Tapada da Ajuda, 1349-017 Lisbon, Portugal; 3 Centre for the Research and Technology Agro-Environmental and Biological Sciences (CITAB), University of Trás-os-Montes e Alto Douro, Quinta de Prados, 5000-801 Vila Real, Portugal; 4 Centro Tecnológico de la Carne de Galicia, Rúa Galicia No 4, Parque Tecnológico de Galicia, San Cibrao das Viñas, 32900 Ourense, Spain; 5 Área de Tecnología de los Alimentos, Facultad de Ciencias de Ourense, Universidad de Vigo, 32004 Ourense, Spain

**Keywords:** rabbit, yellow lupine, white lupine, meat, diet, fatty acids

## Abstract

In Europe, the most appropriate strategy to replace soybean meal (SBM) in animal feed has been the development of diets containing locally produced protein sources. One of these sources is lupine (*Lupinus* spp.). The effect of the total substitution of SBM by white lupine (WL) and yellow lupine (YL) seeds in the diets of growing rabbits and of gender on meat quality and the fatty acids (FA) profile were evaluated. Sixty hybrid weaned rabbits (New Zealand × Californian) (20 rabbits per diet), were fed diets that contained 150 g/kg of SBM (SBMD) and WL (WLD) or YL (YLD) for 35 to 69 days. At the end of this period, 30 rabbits (10 rabbits per diet) were slaughtered to evaluate the carcass and meat characteristics and the FA profile of the longissimus dorsi (LD) muscle. In general, the carcass and meat characteristics (pH and colour) were not affected (*p* > 0.05) by diet or gender. Further, there was no observed effect (*p* > 0.05) of gender on meat FA and on the calculated indexes related to human health. However, diet had an effect (*p* < 0.05) on the FA profile, FA categories, and calculated indexes related to human health. The meat from rabbits fed SBMD presented higher (*p* < 0.05) saturated FA (SFA; 44 vs. 39 g/100 g average on lupine diets) and lower (*p* < 0.05) polyunsaturated FA (PUFA; 24 vs. 28 g/100 g average on lupine diets). Our results showed that SBM may be completely replaced by WL or YL, improving the quality of LD muscle FA in terms of nutritional quality for humans.

## 1. Introduction

For the past ten years, the European Union has been trying to change its dependence on imports of soybean for animal feed. Different legume species, such as lupines (*Lupinus* spp.), faba beans (*Vicia faba*), and peas (*Pisum sativum*), are well adjusted to the Mediterranean soil and climate and have been studied as alternative protein sources for animal feed. Even though these Mediterranean legumes present lower protein contents than soybean meal (SBM), several studies have showen that they could totally or partially replace SBM in lamb [1,2,3], pig [4,5,6], poultry [7,8] and rabbit [9,10] feed.

Lupine seeds are a valuable source of nitrogen and energy [11], with a higher crude protein (CP) content than the other legumes (CP; 280–360 g/kg dry matter, DM, [12]; 290–454 g/kg DM, [13]) and have an interesting ether extract content (EE; 32.8–106 g/kg DM, [13]). Furthermore, lupine seeds present low fibre levels (neutral detergent fibre, NDF; 200–330 g/kg DM, [12]; 189–312 g/kg DM, [13]) and high levels of non-starch polysaccharides (99–332 g/kg DM, [13]) content. Because of their high protein level and their content of fibre and non-starch polysaccharides, lupine is a potentially valuable protein and energy source for ruminant and non-ruminant animals.

Several authors have studied the use of whole or dehulled lupine seeds in the diets of lactating rabbits and for growing/fattening rabbits, and they have shown positive results, with similar values to those obtained from SBM diets. Most studies were conducted with white lupines (WL) [9,14,15,16,17]. In a recent study, Garcia-Santos et al. [18] showed that yellow lupine (YL) is also a suitable dietary protein source for growing rabbits and can totally replace SBM.

The results found on the carcass traits and meat quality of rabbits fed WL are also relevant, indicating that it is an adequate substitute for SBM. Volek and Marounek [9] found a higher dressing-out percentage in rabbits fed a WL diet in comparison with those fed sunflower meal (SFM) or SBM diets. Uhlířová et al. [16] reported a higher chilled and carcass weight in rabbits fed the WL diet than in rabbits fed the SBM diet, whereas the dressing-out percentage was not affected by the diet supplied to the animals. Similarly, Volek and Marounek [19] and Volek et al. [20] did not observe any effect of diet (WL vs. SFM diet and dehulled WL vs. SBM diet, respectively) on the dressing-out percentage of rabbits.

Regarding meat quality, Volek and Marounek [19] observed that the chemical composition of hind leg (HL) meat was not significantly affected by diet (WL vs. SFM diet), and Volek et al. [20] observed that the physical characteristics of the longissimus dorsi (LD) muscle (pH, colour, and cooking losses) were not affected by diet (WL vs. SBM diet), with the exception of the shear force that was higher in animals fed the SBM diet. The effect of WL incorporation in the diets of rabbits was also evaluated by Volek et al. [20]. The results obtained by these authors showed that feeding a diet of dehulled WL significantly decreased the saturated FA (SFA) content, as well as the polyunsaturated FA (PUFA) n − 6/n − 3 ratio and the saturation, atherogenic, and thrombogenic indexes in a rabbit’s HL meat. An increase in monounsaturated FA (MUFA) and in linolenic acid contents was also observed, but no differences were found in PUFA content [20]. These results are consistent with the previous findings of Volek and Marounek [19], who observed that feeding a WL diet compared to an SFM diet to growing rabbits resulted in a lower SFA content, PUFA n − 6/n − 3 ratio, and saturation, atherogenic, and thrombogenic indexes in the HL meat. As far as we know, the effect of incorporating YL in rabbit diets on carcass and meat quality has not yet been studied.

The chemical composition of meat from rabbits, especially fat content and the FA profile, is influenced by sex. The meat of female rabbits presents higher fat content than males slaughtered at 77 and 90 days [21] or at 93 and 105 days [22]. The same authors found no differences between gender in the meat FA composition, with the exception of palmitoleic acid (C16: 1n − 7), which was significantly higher in female rabbits [21,22]. Dalle Zotte et al. [23] observed that gender affected the proportion of total SFA, producing a more favourable SFA/unsaturated FA ratio in females.

The objective of this study was to evaluate the effects of the total replacement of SBM with sweet varieties of WL (*Lupinus albus*, cv. Nacional) or YL (*Lupinus luteus*, cv. Mister) seeds and of gender on the carcass characteristics and meat quality, namely the FA profile of the LD muscle, of growing hybrid rabbits (New Zealand × Californian) of both sexes (50% female and 50% male).

## 2. Materials and Methods

The experiment was conducted in the animal facilities of the University of Trás-os-Montes and Alto Douro (UTAD) at Vila Real (Portugal), and the animals were handled according to Portuguese law on animal welfare in experimental research [24] and the recommendations of the European Group for Rabbit Nutrition [25], as described in Garcia-Santos et al. [18].

### 2.1. Animals and Housing

Sixty weaned hybrid rabbits (New Zealand × Californian) of both sexes (50% female and 50% male) were reared in a building with closed air-conditioning and maintained at 18–23 °C. The day/night cycle was 12/12 h (light from 07:00 to 19:00).

Rabbits weaned at 35 days of age and with similar live weights (LW, 1025.7 ± 118.8 g) were lodged in 30 metabolic cages (50 × 60 × 40 cm; 2 rabbits/cage) and were randomly distributed to three different dietary treatments. The sex distribution was random for all groups.

### 2.2. Experimental Diets

Three diets were formulated to be nearly isonitrogenous and isoenergetic, according to the recommendations for growing rabbits by De Blas and Mateos [26], and they are described in Garcia-Santos et al. [18]. The three experimental diets were: the SBM diet (SBMD) containing 150 g/kg of SBM as the main protein source, and two diets with the complete replacement of SBM by 150 g/kg seeds of WL (WL diet; WLD) or 150/kg YL (YL diet; YLD). The diets were offered in pelleted form throughout the experimental period. The ingredients of the three experimental diets and their chemical compositions are presented in Table 1.

Each experimental diet was randomly assigned to 10 cages. In order to avoid digestive disturbances, a feed restriction (80%) was performed during the first 14 days of the experiment. This restriction was based on recommendations for rabbits during the post-weaning period, and the amount of feed supplied to the animals was planned daily. After this period, the animals were fed ad libitum until the end of the trial. Diets were offered in pelleted form throughout the experimental period. The rabbits had free access to drinking water through nipple drinkers, and during this study, no antibiotics were used in the feed or drinking water. More detailed information on animal handling and diets can be found in Garcia-Santos et al. [18].

### 2.3. Slaughter, Measurements, and Samples Collection

At 69 days of age, 10 rabbits per treatment (of both sexes (average LW 2692 ± 137.9 g)), were slaughtered by sudden cervical dislocation, without fasting, which is the method accepted by Portuguese law on animal welfare in experimental research [24] and as previously stated in Garcia-Santos et al. [18]. The animals were slaughtered under veterinary supervision at the experimental abattoir of the university and did not undergo transport.

The carcasses traits were measured and calculated according to the recommendations of the World Rabbit Science Association [27]. The skin, external genital organs, stomach, and small and large intestines were removed, and the hot carcass was weighed (hot carcass weight, HCW). Carcasses containing the thoracic viscera (thymus, lungs, heart, and oesophagus), liver, kidneys, and head were suspended from the calcaneus tendon for 60 min in a ventilated area and then cooled at 3 °C for 24 h. Afterwards, the carcasses were weighed to acquire the chilled carcass weight (CCW) and the dressing out percentage (CCW/LW) was determined. The chilling loss percentage was calculated as the difference between the HCW and the CCW, relative to the HCW. After remotion and weighing of the liver, head, kidneys, and thoracic viscera, the reference carcass weight was obtained. The perirenal, inguinal, and scapular fat (dissectible fat) of the carcass were removed and weighed. The carcasses obtained were dissected according to the norms of the World Rabbit Science Association [27] and the hind parts, foreparts, and intermediate parts were weighed.

At 24 h post-mortem, the carcass and meat colour were assessed on the surface over the HL (biceps femoris muscle) and on the transversal section of LD muscle, respectively, in accordance with Ouhayoun and Dalle Zotte [28]. The colour was measured at two different sites on both muscles using a Minolta CR-300 Chroma Meter (Minolta Camera, Osaka, Japan) in the CIELAB System [29]: lightness (L*), redness (a*), and yellowness (b*). The values of the colour saturation (chroma, C*) and colour hue (hue, H*) were calculated from the following formulae (CIE 1976): H* = tg − 1(b*/a*) and C* = (a*^2^ + b*^2^) ½.

The ultimate pH (pHu) was measured 24 h post-mortem by directly inserting the electrode into the LD muscle using a pH meter and allowing the temperature setting (pH 91, WTW, Weilheim, Germany).

The LD muscle was dissected between the first and seventh lumbar vertebrae from the two sides of refrigerated carcasses (24 h at 3 °C). Samples of the LD muscle were excised and stored (−20 °C) for determination of the FA and cholesterol contents.

### 2.4. Chemical Analyses

Dried samples of the experimental diets were ground to pass a 1 mm screen and were analysed, according to the AOAC [30] procedures, for DM (934.01), CP (954.01), EE (920.39), and starch (996.11). Fibre fractions (NDF; acid detergent fibre, ADF; acid detergent lignin, ADL) were determined by the detergent procedures of Van Soest et al. [31]. The soluble sugar content was determined by UV-VIS spectrophotometry (UVmin-1240, Shimadzu, Quioto, Japan; 625 nm) according to the anthrone method [32]. These procedures have been previously described in Garcia-Santos et al. [18].

The fatty acids were determined by the procedures described by Argemi-Armengol et al. [33]. The intermuscular and subcutaneous fat was trimmed from the LM samples prior to FA analysis. The FA methyl esters were obtained by transesterification using a 2% (*v*/*v*) methanol/sulfuric acid solution, with heating for 30 min at 80 °C, centrifugation at 3000 rpm for 5 min, and collection of the final supernatant. The analysis of the FA methyl esters was performed in duplicate by gas chromatography with a 30 m × 0.25 mm capillary column and a flame ionization detector (Agilent DB-23; Agilent Technologies, Santa Clara, CA, USA). The applied carrier gas was helium at a flow rate of two mL/min. The oven temperature was programmed to increase at 35 °C per minute between 150 °C and 180 °C and at 5 °C per minute up to 220 °C. A temperature of 250 °C was considered for the injector and detector. The relative percentage of each individual FA in relation to the total FA was considered. The FA were identified by comparison of the retention times with a known standard Supelco^®^37 Component FAME Mix (Supelco, Bellefonte, PA, USA). Thirty-three FA were detected and quantified. The proportion of SFA (C10:0; C12:0; C14:0; C15:0; C16:0; C17:0; C18:0; and C20:0), PUFA (C18: 2n − 6; C18: 3n − 3; C18: 3n − 6; C20: 2n − 6; C20: 3n − 6; C20: 4n − 6; and C22: 5n − 3), and MUFA (C14: 1n − 5; C15: 1n − 5; C16: 1n − 7; C17: 1n − 7; C18: 1n − 7; C18: 1n − 9; C20: 1n − 9; and C22: 1n − 9) was calculated. Short and medium SFA were considered when C < 16, according Panth et al. [34].

To determine the total cholesterol, saponification, extraction, and identification were carried out according to the procedures described by Dominguez et al. [35], with modifications [36,37]. For saponification, 2 g of homogenised sample was placed in a screw Teflon-lined cap tube, and 0.25 g of L-ascorbic acid and 5 mL of saponification solution were added. The air was removed from the reaction by displacement with nitrogen gas, and the sample was vortexed until the ascorbic acid was completely dissolved. The samples were rested for 5 min and then were shaken. The saponification was performed in a shaking water bath (THER-SPIN, Orto Alresa, Madrid, Spain) (200 rpm) at 85 °C for 45 min. The samples were vortexed after 20 min of the saponification process. After saponification, the samples were cooled and, successively, 1.5 mL of distilled water and 3 mL of 25 µg/mL BHT solution in n-hexane were added. At this point, the samples were vigorously vortexed and centrifuged at 1500× *g* for 3 min in order to accelerate phase separation. An aliquot of the upper layer (n-hexane) was transferred into a small screw Teflon-lined cap tube, and a spatle tip of anhydrous sodium sulphate was added. Finally, the tube was briefly shaken, and an aliquot of the n-hexane layer was filtered through a 0.45 µm nylon syringe filter (Filter Lab, Barcelona, Spain) into an amber screw-cap vial with a Teflon septum. The HPLC systems used were an Alliance 2695 model (Waters, Milford, MA, USA) and a 996 Photodiode Array Detector (Waters, Milford, MA, USA). Empower 3TM advanced software (Waters, Milford, MA, USA) was employed in order to control system operation and results management. The analysis of cholesterol was carried out using a normal-phase silica column (Sun Fire TM Prep Silica, 4.6 mm ID × 250 mm, 5 µm particle size; Waters, Milford, MA, USA), with photodiode array detection for cholesterol (208 nm). The solvent (2% *v*/*v* isopropanol in n-hexane) flow rate was 1 mL/min, the run lasted for 17 min, and the temperature of the column oven was adjusted to 30 °C. The content of total cholesterol in foal meat was quantified based on the external standard technique from a standard curve of peak area vs. concentration. The results were expressed as mg of cholesterol/100 g of meat [35,36,37].

### 2.5. Calculations

The atherogenic (AI), thrombogenic (TI), and saturation (S/U) indexes were calculated according to Ulbricht and Southgate [38]. The hypocholesterolemic/hypercholesterolemic ratio (h/H) and desirable FA (DEA) were computed as suggested by Santos-Silva et al. [39]:

AI = (C12:0 + 4 × C14:0 + C16:0)/[ΣMUFA + Σ(n − 6) + Σ(n − 3)]

TI = (C14:0 C16:0 C18:0)/[0.5 ΣMUFA 0.5 Σ(n − 6) + 3 Σ(n − 3) Σ(n − 3)/Σ(n − 6)]

S/U = (C14:0 + C16:0 + C18:0)/ΣMUFA + ΣPUFA

h/H = (C18: 1n − 9 + C18: 2n − 6 + C20: 4n − 6 + C18: 3n − 3 + C20: 5n − 3 + C22: 5n − 3 + C22: 6n − 3)/(C14:0 + C16:0)

DFA = FA unsaturated + C18:0.

To perform the statistical analysis, the JMP version 14 program was used [40] and the general linear model was applied. For all parameters presented, each animal was considered as the experimental unit. Data were submitted to factorial analysis with the diet and gender as the main factors, according to the following model:

Yij = µ + Di + Gj + DiGj + eijk

where:

Yij = dependant variable;
µ = general mean;
Di = fixed effect of diet (i = 1,2,3);
Gj = fixed effect of gender (j = 1,2);
DiGj = interaction effect between diet and gender; and
eijk = residual random error.

Statistical significance was accepted at a *p* value of < 0.05, and a *p* value of between 0.05 and 0.1 was interpreted as a trend. If the diet effects were significant, the Tukey’s multiple comparison test was applied to the compare the means.

## 3. Results

### 3.1. Carcass Characteristics

The effects of diet and gender on the carcass characteristics of 69-day-old rabbits are presented in Table 2.

Regarding the effect of gender on carcass characteristics, no significant differences were observed (*p* > 0.05), and only HCW and CCW showed a tendency to be higher (*p* = 0.090 and *p* = 0.067, respectively, for HCW and CCW) in males. Colour (a*, b*, c*, C*, and H*) was not affected by gender (*p* > 0.05). Carcass parts were also not affected (*p* > 0.05) by gender, with the exception of the hind part, which was lower (*p* = 0.047) in males (29.2 vs. 30 g/100 CCW).

No differences (*p* > 0.05) were observed in the carcass characteristics of 69-day-old rabbits between the diets, although there was a tendency (*p* = 0.054) for rabbits fed the WLD to have a lower slaughter weight (2605 g, 2731 g, and 2739 g for WLD, YLD, and SBMD, respectively). The colour characteristics and carcass parts were not affected by diet (*p* > 0.05).

The interaction effect on the carcass characteristics was non-significant (*p* > 0.05)

### 3.2. Meat Quality and Fatty Acid Profile

The effects of diet and gender on the meat physical characteristics of the LD muscle are presented in Table 3.

No significant differences (*p* > 0.05) were found between sexes for pHu. However, for colour, a significant difference (*p* < 0.05) was found for yellowness (a*; *p* = 0.036), redness (b*; *p* = 0.023), and hue (C*; *p* = 0.006), with higher values for males. For a*, the value found for males was 57% higher than that observed for females (3.31 vs. 5.26). The diet and the interaction diet ×gender had no significant effect (*p* > 0.05) on the physical characteristics (pHu and colour) of the LD muscle of the rabbits.

The effects of diet and gender on FA (g/100 g) and cholesterol (g/100 g) composition of the LD muscle of rabbits at 69 days of age are presented in Table 4. The data show that palmitic (C16:0), oleic (C18:1n − 9) and linolenic (C18: 1n − 6) acids represent more than 60% of the total identified FA.

Gender had no significant (*p* > 0.05) effect on the LD muscle FA profile of the rabbits. However, a tendency for a high stearic (C18:0) acid content (*p* = 0.001) in females was observed (6.92 g/100 g FA and 6.23 g/100 g FA for females and males, respectively).

Diet had an effect (*p* < 0.05) on almost all of the FA presented in Table 4. Palmitic acid was higher in the LD muscle of rabbits fed the SMBD (32.01 g/100 g FA; *p* < 0.05), though no significant differences (*p* > 0.05) between WLD and YLD (average 28.38 g/100 g FA) were found. Meat from the rabbits fed the WLD presented the highest (*p* < 0.05) content of oleic (27.5 g/100 g FA and 24.8 and 24.3 g/100 g FA for SBMD and YLD, respectively) and α-linolenic (3.87 for WLD and 2.6 g/100 g FA for SBMD and YLD) acids. The cholesterol content of the LD muscle was affected by diet (*p* = 0.02), with the lowest (*p* < 0.05) value of YLD (25.5 g/100 g).

The interaction effect on the LD muscle FA profile of the rabbits was not significant (*p* > 0.05).

The FA categories and the nutritional indexes related to human health of the LD muscle of rabbits at 69 days of age are presented in Table 5. No effects of gender or interaction of diet ×gender (*p* > 0.05) were observed in any FA categories or in the calculated indexes related to human health presented in Table 5.

In general, diet had an effect (*p* < 0.05) on FA categories and indexes related to human health of the LD muscle of rabbits at 69 days of age (Table 5). The total SFA determined in this muscle was lower (*p* < 0.05) on lupine diets (38.7 and 40.5 g/100 g FA on WLD and YLD, respectively, and 44.2 g/100 g FA on SMBD). The same was observed for all categories of SFA (short and media chain saturated fatty acids, *p* = 0.018; long-chain saturated fatty acids, *p* < 0.001; and odd-numbered FA, *p* = 0.0003). The opposite was observed in total PUFA content, which was higher (*p* < 0.05) on lupine diets (27.4 and 29.0 g/100 g FA and 24.21 g/100 g FA, respectively for WLD, YLD, and SMBD). The n − 6/n − 3 ratio for LD meat presented the highest (*p* < 0.05) value for rabbits fed the YLD (8.8), followed by the SBMD (7.1) and then the WLD (5.4), which had the lowest value.

The lower levels of SFA and higher levels of PUFA in meat from the rabbits fed the lupine diets promoted a higher (*p* < 0.05) DFA in the meat from the rabbits fed these diets (67.7 g/100 g FA and 62.9 g/100 g FA for WLD and YLD, respectively). The h/H index was significantly higher (*p* < 0.05) in rabbits fed the lupine diets, about 30% higher than that observed in those fed the SBMD. The LD meat of the rabbits fed the WLD presented the lowest (*p* < 0.05) TL value (0.886), followed by YLD (1.033) and then by SBMD, which had the highest value (1.190). The TI was 58% lower (*p* < 0.05) in rabbits fed the WLD than that observed in rabbits fed the SBMD.

## 4. Discussion

### 4.1. Carcass Characteristics

The carcass characteristics and meat quality were not affected by gender, although there was a tendency for males to have a high HCW and CCW. Similar results were found by Ortiz Hernández and Rubio Lozano [41] for the CCW, dressing percentage, and drip losses of the carcasses of rabbits slaughtered at 70 days, although the chilling time used by these authors was shorter than ours (6 vs. 24 h). No effects on the carcass characteristics were observed by Trocino et al. [42] and Yalçın et al. [43] for rabbits slaughtered at 77 days. Luzi et al. [44] found no effect of sex on the CCW and dressing percentage when rabbits were slaughtered at 90 days, but they observed a difference when rabbits were slaughtered at 120 days of age. In most species, males have a higher growth potential than females, and when rabbits are slaughtered prior to reaching adult weight, the differences are rarely noticeable [41]. This might be the reason for the lack of difference observed between genders on the carcass characteristics.

The incorporation of lupine in the diets of growing rabbits had no effect on the carcass characteristics. In addition, Uhlírová et al. [16] and Volek et al. [20] found that the dressing-out percentage was not affected by diet in rabbits fed a WL diet and an SBM diet. On the contrary, Volek and Marounek [9] found a higher (58.7%) dressing-out percentage in rabbits fed a WL diet in comparison to those fed an SBM (57.3%) diet. According to Volek [20], the “contradictory results regarding the dressing-out percentage may be related to a full digestive tract and skin weight, as well as slaughter weights of rabbits used for the determination of carcass characteristics in different experiments”.

The live weight at slaughter is one of the main factors affecting carcass quality [45] and, therefore, it was expected that in our study there would be no effect of diet or gender on carcass characteristics since slaughter weight was not different between the diets or genders.

### 4.2. Meat Quality and Fatty Acids Profile

The meat physical properties (colour and ultimate pH) measured in the LD muscle were not affected by gender or diet, and no interaction between diet and gender was observed. Most studies have described no significant difference between females and males in the ultimate pH (pH 24 h after slaughter) in the LD muscle of animals slaughtered between 70 and 120 days of age [42,46,47,48,49]. According to Składanowska-Baryza et al. [50], slaughter conditions and transport time are factors that affect the pH of the meat. These conditions were the same for all animals, and a different pH would not be expected.

The meat colour parameters which were assessed 24 h post-mortem were not affected by gender. Nonetheless, a trend toward lower a*, b*, and hue (H*) values in females compared to males was observed. The a* value in males was 57% higher than the values observed in females, indicating redder meat in males than in females. Cavani et al. [46] also observed that males had significantly higher a* and C* values (*p* < 0.01 for a* and *p* < 0.05 for H*) in the LD muscle at 24 h post-mortem. Dalle Zotte et al. [48] found that the b* value was higher (*p* < 0.05) in the LD muscle of females than in males at 24 h post-mortem. However, most studies showed no effect of sex on meat (LD muscle) colour at 24 h after slaughter, with males presenting high values for a* and b* [22,42,49,51]. As stated above, for carcass characteristics, this result may depend on the slaughter age of the rabbits, as the gender differences become more evident when a rabbit’s age approaches puberty.

No differences due to gender were found in the FA composition of LD muscle, with the exception of the content of stearic acid (C18:0), which was higher in the female rabbits compared to the male rabbits. The effect of gender in the FA profile of rabbit meat varies greatly among studies. The effect of gender on the FA content of meat (LD muscle) was noted only for capric acid (C10:0, *p* = 0.02 [52]) or oleic acid (C18: 1n − 9, *p* = 0.01 [53]), which were higher in males. Dalle Zotte et al. [23] observed an effect of gender on three FA of the HL muscle, with C16:0 and C20: 3n − 6 higher (*p* < 0.05) in males and C18:3n-3 higher (*p* < 0.05) in females. The FA categories (SFA, MUFA, and PUFA) were not affected by sex, which agrees with the results obtained by Gasperlin et al. [21] and Polak et al. [22] on rabbit meat (LD + abdominal wall + HL) and those of North et al. [53] and Daszkiewicz and Gugołek [52] on LD muscle. Our data also showed no effect of gender on the calculated nutritional indexes related to human health, which is in agreement with the studies of other authors [21,22,52,53].

The substitution of SBM by WL or YL promoted an effect on almost all FA content. The meat of the rabbits fed lupine diets was characterised by a more desirable FA composition and a higher concentration of MUFA and PUFA. We were not able to find any work comparing an SBM diet with a WLD or YLD. However, Kowalska et al. [54] also found that WL, at a level of 8 g/100 g, together with 10 g/100 g of rapeseed meal and 6g/100 g of peas, significantly increased the PUFA and MUFA of rabbit’s muscles and decreased the SFA content compared with an SBM diet (15 g/100 g). The FA of lupine, although variations exist among the varieties, is characterised to present high amounts of MUFA (mainly C18: 1n − 9) and PUFA (C18: 2n − 6, C18: 3n − 3) and low amounts of SFA, which explains the differences found in the FA profiles of rabbit meat. Thus, the FA in meat reflects the changes in the FA of the diet.

The PUFA n − 6/n − 3 ratio and the nutritional indexes related to human health were improved in rabbits fed the WLD and the YLD, indicating that lupine diets yielded LD meat that may provide a nutritional benefit to humans. Our results confirm the previous findings of Volek and Marounek [19] and Volek et al. [20] in the HL muscle of rabbits fed, respectively, diets of SFM or WL or diets of SBM or dehulled WL. In both works, the incorporation of WL in rabbit diets improved the PUFA n − 6/n − 3 ratio, as well as the indexes related to human health.

Rabbits fed the two lupine diets studied, the WLD and YLD, presented non-significant differences for almost all of the FA and the FA categories. The same was observed for all the indexes related to human health analysed, except for the TI, which was lower in the LD muscle of the rabbits fed the WLD.

## 5. Conclusions

From the present study, it follows that SBM may be completely replaced with WL or YL (150 g/diet) in the balanced diets of growing rabbits, supplemented with amino acids, and having no negative effect on the carcass characteristics and meat physical properties (pH24 and colour). However, the total substitution of SBM by WL or YL improved the quality of the LD muscle FA in terms of its nutritional quality for humans. Thus, as a general conclusion, the use of lupine as an ingredient in the diet of rabbits has a positive effect on its nutritional quality without affecting the productivity of the animals, and it is an important strategy for reducing dependence on soybean meal for animal feed.

## Figures and Tables

**Table 1:** Ingredients and chemical compositions (g/kg as-fed basis) of the experimental diets (Adapted with permission from Ref. [18]. 1 August 2021, Elsevier).

	Diet		
	SBMD	WLD	YLD
Ingredients			
Soybean meal	150	0	0
White lupine seed	0	150	0
Yellow lupine seed	0	0	150
Wheat	149	21	92
Wheat bran	155	350	350
Sugar cane molasses	10	10	10
Lucerne	189	263	127
Grape seed meal	50	50	50
Wheat straw	120	32	107
Beet pulp	150	80	80
Calcium carbonate	7	6	14
Monocalcium phosphate	7	4	4
Salt (NaCl)	4	4	5
Sepiolite	0	18	0.2
Mineral-vitamin premix ^a^	10	10	10
Biolys ^b^	0	0	0.35
Chemical composition			
Dry matter	916	913	910
Crude protein	161	164	163
Ether extract	35	34	42
NDF	346	344	364
ADF	217	227	212
ADL	52	64	55
Soluble sugars	34	37	35
Starch	124	81	120
Digestible crude protein ^c^	120	123	123
Digestible energy (MJ/kg) ^c^	9.7	9.7	9.7
Digestible lysine ^c^	7.5	6.5	6.5
Digestible methionine + cysteine ^c^	3.8	3.0	2.8

SBMD, soybean meal diet; WLD, white lupine diet; YLD, yellow lupine diet; NDF, neutral detergent fibre expressed exclusive of residual ash; ADF, acid detergent fibre expressed exclusive of residual ash; ADL, acid detergent lignin. ^a^ Mineral and vitamin mixture (10 g/kg) providing the following nutrients per kg of feed: vitamin A, 10,000 IU; vitamin D3, 1080 U; vitamin E, 36 mg; vitamin K, 1 mg; vitamin B1, 2 mg; vitamin B2, 6 mg; vitamin B6, 2 mg; vitamin B12, 10 mg; niacinamide, 50 mg; Ca- pantothenate, 20 mg; folic acid, 5 mg; Fe, 78 mg; Cu, 14 mg; Co, 0.5 mg; Mn, 20 mg; Zn, 60 mg; Se, 0.05 mg; I, 1.1 mg; and choline chloride, 260 mg. ^b^ Biolys composition: lysine, 5 g/kg; methionine, 1 g/kg; methionine + cysteine, 1.6 g/kg; threonine, 2.8 g/kg; tryptophan, 0.4 g/kg; arginine, 5.7 g/kg, valine, 3.7 g/kg; isoleucine, 3 g/kg; and leucine, 4.9 g/kg. ^c^ values determined by the supplier (calculated using CVB (2018) methodologies, based on the chemical analysis of the raw materials).

**Table 2:** Effects of diet and gender on the carcass characteristics of 69-day-old rabbits (*n* = 10 per treatment).

	Diet (D)			Gender (G)			*p* Value		
	SBMD	WLD	YLD	F	M	SEM	D	G	D × G
Slaughter weight (SW, g)	2739	2605	2731	2672	2738	25.2	0.054	0.123	0.731
Hot carcass weight (g)	1693	1614	1670	1644	1694	16.2	0.135	0.090	0.284
Hot dressing (g/100g SW)	61.8	61.9	61.2	61.5	61.9	0.28	0.737	0.713	0.273
Chilled carcass weight (CCW, g)	1658	1579	1623	1603	1659	16.5	0.185	0.067	0.240
Chilling loss (g/100g)	2.09	2.13	2.20	1603	2.07	0.070	0.876	0.484	0.854
Chilled dressing (g/100g SW)	60.5	60.6	59.4	60.0	60.6	0.25	0.405	0.343	0.058
Carcass parts (g/100g of CCW)									
Head	7.98	7.97	7.96	8.04	7.79	0.097	8.66	0.221	0.479
Liver	7.69	7.52	7.05	7.40	7.47	0.159	0.242	0.866	0.369
Kidneys	1.20	1.26	1.21	1.22	1.23	0.016	0.219	0.809	0.405
Thymus, lungs, and heart	2.05	2.24	2.15	2.17	2.09	0.380	0.054	0.102	0.135
Total fat	2.89	2.83	2.91	2.83	2.97	0.083	0.688	0.587	0.261
Scapular fat	0.66	0.74	0.66	0.65	0.76	0.032	0.667	0.187	0.776
Perineal fat	1.51	1.52	1.64	1.58	1.50	0.068	0.333	0.398	0.169
Inguinal fat	0.72	0.57	0.62	0.60	0.71	0.048	0.409	0.234	0.839
Hind part	29.7	29.7	29.0	30.0	29.2	0.180	0.685	0.047	0.045
Forepart	28.5	28.6	29.4	28.7	29.3	0.240	0.097	0.191	0.187
Intermediate part	19.9	19.8	20.2	20.0	19.9	0.190	0.725	0.788	0.894
CIE carcass *									
L*	47.5	46.9	48.0	47.6	47.0	0.59	0.675	0.510	0.254
a*	6.3	7.4	5.9	5.94	7.83	0.65	0.788	0.196	0.612
b*	8.3	10.5	8.5	8.99	9.36	0.56	0.413	0.823	0.491
Chroma (C*)	12.2	12.8	10.6	11.0	12.7	0.80	0.589	0.364	0.417
Hue (H*)	1.07	0.96	0.95	0.99	0.95	0.029	0.764	0.590	0.219

SBMD, soybean meal diet; WLD, white lupine diet; YLD, yellow lupine diet; F, Female; M, Male; SEM, standard error of mean. * CIE determined at the surface of the biceps femoris muscle. Each animal was considered an experimental unit for this experiment.

**Table 3:** Effects of diet and gender on the meat physical characteristics of the longissimus dorsi muscle of rabbits (*n* = 10 per treatment).

	Diet (D)			Gender (G)			*p* Value		
	SBMD	WLD	YLD	F	M	SEM	D	G	D × G
Ultimate pH	5.95	5.97	5.92	5.96	5.92	0.036	0.843	0.551	0.873
CIE									
L*	46.6	45.7	44.3	45.6	45.4	0.59	0.593	0.867	0.801
a*	3.48	3.77	4.49	3.34	5.26	0.390	0.366	0.036	0.576
b*	11.0	11.2	10.9	10.7	11.8	0.21	0.841	0.023	0.921
Chroma (C*)	11.5	11.9	11.9	11.2	13.2	0.30	0.788	0.006	0.690
Hue (H*)	1.27	1.26	1.20	1.27	1.17	0.026	0.424	0.09	0.720

SBMD, soybean meal diet; WLD, white lupine diet; YLD, yellow lupine diet; F, Female; M, Male; SEM, standard error of mean. Each animal was considered an experimental unit for this experiment.

**Table 4:** Effects of diet and gender on the major fatty acids and cholesterol (g/100 g identified FA) composition of the longissimus dorsi muscle of rabbits at 69 days of age (*n* = 10 per treatment).

	Diets (D)			Gender (G)			*p* Value		
	SBMD	WLD	YLD	F	M	SEM	D	G	D × G
SFA									
C10:0	0.117	0.086	0.132	0.1086	0.1189	0.01086	0.327	0.532	0.804
C12:0	0.387	0.307	0.386	0.3501	0.3850	0.01803	0.148	0.233	0.740
C14:0	3.035 a	2.495 b	2.832 ab	2.7966	2.7667	0.0676	0.006	0.654	0.451
C15:0	0.5837 a	0.501 b	0.509 b	0.5467	0.5053	0.01108	0.013	0.181	0.807
C16:0	32.010 a	27.82 b	28.93 b	29.7185	29.3050	0.33970	<0.001	0.668	0.413
C17:0	0.674 a	0.570 b	0.580 b	0.6292	0.5588	0.01399	0.006	0.042	0.570
C18:0	7.085	6.416	6.651	6.9247	6.2348	0.11532	0.123	0.001	0.834
C20:0	0.139 b	0.205 a	0.208 a	0.1829	0.1867	0.00692	<0.001	0.340	0.055
MUFA									
C14: 1n − 5	0.244 a	0.1682 b	0.230 ab	0.2125	0.2183	0.01146	0.034	0.478	0.691
C15: 1n − 5	0.423 a	0.332 b	0.296 b	0.3594	0.3282	0.01664	0.018	0.446	0.222
C16: 1n − 7	3.834 a	2.836 b	3.417 ab	3.3395	3.4178	0.1275	0.011	0.365	0.751
C17: 1n − 7	0.289 a	0.209 b	0.233 b	0.2484	0.2329	0.00767	<0.001	0.933	0.512
C18: 1n − 7	1.224 b	1.478 a	1.111 b	1.2408	1.3427	0.03809	<0.001	0.345	0.525
C18: 1n − 9	24.78 b	27.50 a	24.27 b	25.2018	26.2625	0.33732	<0.001	0.161	0.624
C20: 1n − 9	0.362 c	0.821 a	0.466 b	0.5210	0.6158	0.03921	<0.001	0.581	0,480
C22: 1n − 9	0.216 ab	0.232 a	0.182 b	0.2158	0.1980	0.00733	0.002	0.094	0.113
PUFA									
C18: 2n − 6	19.65 c	21.73 b	24.60 a	22.0325	21.9234	0.46914	<0.001	0.529	0.771
C18: 3n − 3	2.606 b	3.875 a	2.653 b	2.9932	3.1666	0.11689	<0.001	0.694	0.433
C18: 3n − 6	0.084 a	0.060 b	0.072 ab	0.0756	0.0630	0.00350	0.017	0.269	0.322
C20: 2n − 6	0.222 b	0.268 a	0.273 a	0.2158	0.2556	0.00722	0.009	0.625	0.779
C20: 3n − 6	0.145	0.122	0.135	0.1401	0.1196	0.00425	0.226	0.049	0.564
C20: 4n − 6	0.895	0.863	0.801	0.8887	0.7789	0.03140	0.619	0.119	0.730
C22: 5n − 3	0.216 ab	0.233 a	0.1826 b	0.2158	0.1980	0.00733	0.002	0.094	0.113
Cholesterol	28.60 a	28.46 a	25.56 b	27.63	27.35	0.470	0.020	0.694	0.724

SBMD, soybean meal diet; WLD, white lupine diet; YLD, yellow lupine diet; F, Female; M, Male; SEM, standard error of mean; SFA, saturated fatty acids; MUFA, monounsaturated fatty acids; PUFA, polyunsaturated fatty acids. a, b, c Means with different letters in the same row differed (*p* < 0.05). Each animal was considered an experimental unit for this experiment.

**Table 5:** Effects of diet and sex on mean contents (g/100 g FA) of fatty acid categories and nutritional indexes of the longissimus dorsi muscle rabbits at 69 days of age (*n* = 10 per treatment).

	Diets (D)			Gender (G)			*p* Value		
	SBMD	WLD	YLD	M	F	SEM	D	G	D × G
SFA									
Total	44.21 a	38.67 b	40.47 b	41.47	40.29	0.502	<0.001	0.590	0.548
SCMSFA	4.182 a	3.442 b	3.923 b	3.857	3.831	0.103	0.018	0.645	0.508
LCSFA	40.03 a	35.23 b	36.55 b	37.62	36.46	0.424	<0.001	0.360	0.613
ONFA	2.040 a	1.679 b	1.679 b	1.848	1.686	0.0419	0.0003	0.105	0.938
MUFA	31.66 ab	33.88 a	30.54 b	31.65	32.92	0.380	0.0013	0.145	0.926
PUFA									
Total	24.12 b	27.44 a	28.98 a	26.88	26.78	0.496	<0.001	0.414	0.790
N − 3	2.982b	4.289 a	2.977 b	3.37	3.52	0.128	<0.001	0.499	0.423
N − 6	21.07 b	23.12 b	25.97 a	23.46	23.23	0.473	<0.001	0.427	0.812
N − 6/n − 3	7.086 b	5.404 c	8.753 a	7.18	6.85	0.2610	<0.001	0.859	0.159
Indexes related to human health									
AI	0.8011 a	0.6228 b	0.6846 b	0.6846	0.7099	0.0171	<0.001	0.877	0.491
TI	1.190 a	0.886 c	1.033 b	1.033	1.053	0.0260	<0.001	0.772	0.529
h/H	0.6747 b	0.8905 a	0.9003 a	0.9003	0.8199	0.0248	<0.001	0.478	0.564
S/U	0.7566 a	0.5998 b	0.6463 b	0.6463	0.6775	0.0142	<0.001	0.585	0.562
DFA (g/100 g FA)	62.87 b	67.74 a	66.18 a	65.45	65.94	0.466	<0.001	0.706	0.464

SFA, saturated fatty acid; SCMSFA, short and media chain saturated fatty acids; LCSFA, long-chain saturated fatty acids; ONFA, odd-numbered fatty acids; MUFA, monounsaturated fatty acids, PUFA; polyunsaturated fatty acids, AI, atherogenic index; TI, thrombogenic index; h/H, hypocholesterolemic/hypercholesterolemic; S/U, saturation index; DFA, desirable fatty acids. a, b, c Means with different letters in the same row differed (*p* < 0.05). Each animal was considered an experimental unit for this experiment.

## Data Availability

All data are presented in the manuscript. However, the data may be required from the authors via email.
